# Parameters of heart rate variability in the critically ill subjects with different disease conditions

**DOI:** 10.1186/2197-425X-3-S1-A214

**Published:** 2015-10-01

**Authors:** M Omerbegovic

**Affiliations:** University Clinical Centre Sarajevo, Anaesthsia and ICU, Sarajevo, Bosnia and Herzegovina

## Intr

Heart rate variability has been recognized as a parameter that partly describes autonomic nervous system modulation of cardiovascular system.Analysis of heart rate variability has been proposed as clinically important as predictive and monitoring factor in subjects with different cardiac disease conditions and in subjects who suffer from diabetes mellitus.

Despite numerous clinical and experimental trials on the topic of heart rate variability in the setting of intensive care medicine there is a lack of appropriate technological facilites for routine monitoring and analysis of this phenomenon in everyday clinical practice.

## Objectives

Assessment of the parameters of heart rate variability in critically ill patients with different disease condition.

## Methods

Recordings of forty-two consecutive patients with different disease conditions admitted to intensive care were included in the observational trial. Electrocardiogram was recorded in the periods after admission and primary stabilization of the general condition, and afterwards during the period of stay in intensive care every day (first week of stay). After recording, analysis of short-term segments of electrocardiograms was performed by means of software packages for heart rate vaiability analysis (HrvFreq version 4.0, ©2006 and Kubios HRV version 2.1,2012). Linear parameters in time domain (mean RR, mean HR, SDNN) and parameters in frequency domain (TP, LF,HF,LF/HF) and parameters of Poincaré plot (SD1,SD2) were analysed.

## Results

Statistical analysis of the data obtained was performed by software package IBM SPSS version 20. Diversity of the clinical conditions of the patients and different medications that were used on regular bases limited statistical analysis to only descriptive statistics. Results of assessment of different parameters of heart rate variability in the setting of ICU, in small group of subjects with different comorbid states are presented in this paper.

## Conclusions

Subjects in critical state for different disease conditions or after trauma had different patterns of alterations of heart rate variability. In this small group of patients, despite considerable variations in reduction of different parameters of heart rate variability, alterations of heart rate variability were the most pronounced in subjects who had also had developed acute coronary syndrome before admission to the hospital.
